# Knowledge attitude and practice of older adults rheumatoid arthritis patients regarding disease management in a cross-sectional study

**DOI:** 10.3389/fpubh.2026.1763566

**Published:** 2026-03-09

**Authors:** Yue Gou, Xuedan Shi, Ying Ouyang, Yan Wu, Rong He, Zhi Wan, Qibing Xie

**Affiliations:** 1 Department of Outpatient, West China Hospital, Sichuan University/West China School of Nursing, Sichuan University, Chengdu, China; 2 Department of Outpatient, West China Hospital, Sichuan University, Chengdu, China; 3 Medical Affairs Department of Medical Affairs Division, West China Hospital, Sichuan University, Chengdu, China; 4 The Second People's Hospital of Yibin, Yibin, China; 5 Department of Rheumatology and Immunology, West China Hospital, Sichuan University, Chengdu, China

**Keywords:** cross-sectional study, disease management, older adults, knowledge, attitude, practice, rheumatoid arthritis, structural equation modeling

## Abstract

**Background:**

This study aimed to assess the knowledge, attitude, and practice (KAP) of older adults patients with rheumatoid arthritis (RA) regarding disease management.

**Methods:**

This multicenter cross-sectional study enrolled older adults patients with RA between November and December 2024 in Sichuan Province, China. Self-administered questionnaires were utilized to collect demographic information and evaluate KAP scores.

**Results:**

A total of 488 participants were included in the study, with 72.54% aged 60–69 years and 70.29% female. The mean KAP scores were 10.72 ± 4.68 (possible range: 0–20), 20.11 ± 2.31 (possible range: 6–30), and 25.05 ± 6.10 (possible range: 7–35), respectively. Spearman correlation analysis revealed that knowledge positively correlated with both attitude (*r* = 0.1293, *p* = 0.0042) and practice (*r* = 0.5037, *p* < 0.001), while attitude positively correlated with practice (*r* = 0.2066, *p* < 0.001). The structural equation model showed that knowledge directly affected attitude (*β* = −0.56, *p* < 0.001) and practice (*β* = 0.61, *p* < 0.001), while also indirectly affected practice through attitude mediation (*β* = 0.52, *p* < 0.001). Furthermore, attitude demonstrated a direct effect on practice (*β* = −0.93, *p* < 0.001).

**Conclusion:**

This study found that older adults patients with RA generally exhibited insufficient knowledge and predominantly negative attitudes toward disease management, although many reported relatively proactive behaviors in managing their condition. Targeted educational interventions are necessary to improve knowledge and attitudes, which may further enhance disease management practices among older adults RA patients.

## Introduction

Rheumatoid arthritis (RA) is a chronic, multisystem autoimmune disorder primarily characterized by destructive synovitis and progressive joint damage. Its clinical manifestations include polyarthritis affecting the small joints of the hands and feet, morning stiffness, and various systemic symptoms, all of which significantly impair patients’ joint function and overall quality of life (1). In addition to joint-related manifestations, both RA and its pharmacological treatments are associated with an increased risk of comorbidities, particularly cardiovascular complications, which further increase the overall disease burden and impose substantial social and economic impacts on patients (2–4). Recent data indicate that the global prevalence of RA is approximately 460 per 100,000 individuals (5), with notably higher rates observed among older populations (6–8). In China, the prevalence of RA is estimated to be around 0.42% in the adult population, with higher rates reported among women and older adults individuals (9). Additionally, data from the Chinese Registry of RA (CREDIT) reported a prevalence of 0.42%, corresponding to over 5 million patients by 2013 (10). Other epidemiological studies in mainland China have documented RA prevalence ranging from 0.2 to 0.93% (11). These findings highlight that RA represents a significant public health concern in China, particularly among older populations.

The treatment of RA has been transformed by the introduction of targeted therapies and biological agents, particularly tumor necrosis factor (TNF) inhibitors and Janus kinase (JAK) inhibitors (12). Furthermore, contemporary treatment protocols emphasize the importance of early diagnosis and intervention, along with regular monitoring of disease activity using validated assessment tools (13). A “treat-to-target” approach, which integrates pharmacological interventions and lifestyle modifications, has emerged as the gold standard in managing RA (14). However, the effectiveness of these advanced treatments is heavily contingent upon patients’ adherence to prescribed regimens, a challenge that is especially pronounced among older adults patients managing multiple health conditions (15, 16).

The Knowledge, Attitude, and Practice (KAP) survey is a diagnostic research tool designed to evaluate individuals’ understanding, beliefs, and behaviors regarding a specific topic. In the context of health literacy, it operates on the premise that knowledge shapes attitudes, which in turn influence behaviors. This framework is particularly relevant for examining self-management practices among RA patients (17). Older adults individuals with RA encounter unique challenges in disease self-management due to issues such as polypharmacy and limited awareness of their condition (18). Research indicates that up to 50% of RA patients discontinue prescribed cardiovascular medications within the first year, highlighting the difficulties in achieving long-term adherence to treatment regimens (19). However, most existing studies have primarily focused on general RA populations, with limited attention given to the specific challenges faced by older adults patients (20). Therefore, this study aimed to assess the KAP related to disease management among older adults patients with RA and to explore the interrelationships among these factors using correlation analysis and structural equation modeling (SEM).

## Materials and methods

### Study design and participants

This multicenter cross-sectional study was conducted between November and December 2024, targeting older adults patients with RA from 10 hospitals in Sichuan Province, China (Supplementary Table S1). The study was approved by the Medical Ethics Committee of West China Hospital, Sichuan University (Approval number: #2024-2179), and all participants provided written informed consent prior to participation.

The inclusion criteria included: (1) a confirmed diagnosis of RA. Diagnosis of RA was confirmed through review of the patients’ outpatient electronic medical records and/or inpatient medical records documented in the hospital information system; (2) age 60 years or older; and (3) willingness to participate and provide written informed consent. Patients exhibiting cognitive impairments or sensory disabilities that interfered with verbal communication and comprehension were excluded.

### Questionnaire

The questionnaire was developed based on a review of relevant literature (21). Following the initial draft, a preliminary survey was conducted, yielding 38 valid responses. Reliability analysis demonstrated satisfactory internal consistency, with a Cronbach’s alpha coefficient of 0.812.

The finalized questionnaire, administered in Chinese, was structured into four sections: demographic information, knowledge, attitude, and practice (Supplementary material). Demographic information included age (60–69 years, ≥70 years), gender (male, female), place of residence (rural, urban, suburban), education level (primary school or below, junior high school, high school/technical school, associate degree, bachelor’s degree or above), type of employment (long-term stable job, temporary job, freelance, unemployed, retired, other), per capita household income (<2,000; 2,000–4,999; 5,000–9,999; ≥10,000 CNY), duration of RA (<1 year, 1–3 years, 3–5 years, 5–10 years, >10 years; defined as the time from symptom onset to the completion of the questionnaire), family history of RA (yes, no), treatment type (no treatment, medication therapy, surgery, rehabilitation and physiotherapy, other), stage of RA (early stage, active stage, remission stage, unsure), and type of medical insurance (provincial insurance, municipal insurance, new rural cooperative medical scheme, other). The knowledge dimension comprised 10 items, each scored as “Understand” (2 points), “Partially understand” (1 point), or “Do not understand” (0 points), resulting in a total possible score range of 0–20. The attitude dimension included six items evaluated using a 5-point Likert scale. Positive items were scored from “Strongly agree” (5 points) to “Strongly disagree” (1 point), while negatively worded items were reverse scored. The total score range for this section was 6–30. The practice dimension consisted of seven items, also measured on a 5-point scale ranging from “Never” (1 point) to “Always” (5 points), with a total possible score range of 7–35. For analysis, scores exceeding 70% of the total possible range in the KAP dimensions were categorized as indicative of good knowledge, positive attitude, and proactive practice, respectively (22).

### Questionnaire distribution and quality control

Based on the latest administrative division of Sichuan Province, this study initially aimed to include five secondary or higher-level hospitals from each of the five economic regions: the Chengdu Plain Economic Zone, the Northeastern Sichuan Economic Zone, the Southern Sichuan Economic Zone, the Northwestern Sichuan Ecological Demonstration Zone, and the Panxi Economic Zone. The authors contacted hospitals via email and phone, receiving responses from 15 hospitals. To ensure comparability regarding the prevalence of RA, the development of RA-related departments, and hospital classification, two hospitals from the Northwestern Sichuan Ecological Demonstration Zone, two from the Southern Sichuan Economic Zone, and two from the Panxi Economic Zone were excluded. Ultimately, 10 hospitals, including the authors’ affiliated hospital, participated in the study.

As a multicenter study, the research team collaborated with the heads of the selected hospitals to organize standardized training for hospital staff involved in the survey. A convenience sampling method was used to recruit RA patients aged 60 years and above from these 10 hospitals. During the survey, trained investigators conducted face-to-face interviews using standardized instructions, ensuring consistency in data collection. The questionnaire was administered via Wenjuanxing (an online survey platform), with investigators scanning the QR code using WeChat on their mobile devices and guiding patients through each question. Questionnaires completed in less than 60 s and those with inconsistent responses (such as indicating “No” for medical insurance in Question 7 of the demographic section but providing specific types of insurance in Question 12) were deemed unreliable.

### Sample size calculation

Sample size was calculated using the formula for cross-sectional studies (23): *α* = 0.05, $\;n={\left(\frac{Z_{1-\alpha /2}}{\delta }\right)}^{2}\times p\times (1-p)$ where $Z_{1-\alpha /2}$ = 1.96 when *α* = 0.05, the assumed degree of variability of *p* = 0.5 maximizes the required sample size, and *δ* is the admissible error (set to 5% in this case). The theoretical sample size was 480, with an extra 20% added to account for potential participant dropout.

### Statistical analysis

Statistical analysis was conducted using STATA 17.0 (StataCorp, College Station, TX, USA). Continuous variables were expressed as mean ± standard deviation (mean ± SD), and normality was assessed prior to analysis. For normally distributed variables, independent samples t-tests and one-way analysis of variance (ANOVA) were used. For non-normally distributed variables, the Mann–Whitney U test was used. Categorical variables were presented as frequencies and percentages (*n*, %). Internal consistency reliability of the KAP constructs was evaluated using Cronbach’s *α* and composite reliability (CR). Convergent validity was assessed using average variance extracted (AVE). Discriminant validity was examined using the Fornell–Larcker criterion by comparing the square root of AVE for each construct with inter-construct correlations. Confirmatory factor analysis (CFA) was conducted to examine the factor structure of the KAP constructs. Spearman correlation analysis was performed to examine relationships among KAP scores. SEM was conducted to evaluate three hypothesized pathways among the latent constructs of knowledge, attitude, and practice: (H1) knowledge directly influences attitude, (H2) knowledge directly influences practice, and (H3) knowledge indirectly influences practice through attitude. Spearman correlation analysis was used to examine unadjusted bivariate associations, whereas SEM was applied to estimate conditional relationships among latent constructs within a multivariable framework that accounts for shared variance among variables. Model fit was assessed using standard indices, including the Root Mean Square Error of Approximation (RMSEA), Standardized Root Mean Square Residual (SRMR), Tucker–Lewis Index (TLI), and Comparative Fit Index (CFI) to ensure the adequacy of the hypothesized model. A two-sided *p*-value of less than 0.05 was considered statistically significant throughout the analysis.

## Results

### Basic information

Initially, a total of 630 participants were recruited. After excluding 95 cases with response times <60 s, 1 outlier, and 59 with logical inconsistencies, the final dataset comprised 488 valid responses. The majority of participants were aged 60–69 years (72.54%), female (70.29%), and from urban areas (43.65%). Most had a primary school education or below (34.63%) and a household income of 2,000–4,999 CNY (45.08%). Regarding disease characteristics, 133 (27.25%) had RA for more than 10 years, and 218 (44.67%) were in the remission stage. In terms of treatment, the majority (447, 91.6%) received medication therapy, while 140 (28.69%) underwent rehabilitation and physiotherapy, 41 (8.4%) had surgical interventions, and 65 (13.32%) received other forms of treatment. Only 19 (3.89%) reported receiving no treatment. The mean KAP scores were 10.72 ± 4.68 (knowledge), 20.11 ± 2.31 (attitude), and 25.05 ± 6.10 (practice) (Table 1).

**Table 1:** Demographic characteristics and KAP scores.

*N* = 488	*N* (%)	Knowledge score		Attitude score		Practice score	
		Mean ± SD	*P*	Mean ± SD	*P*	Mean ± SD	*P*
Total score		10.72 ± 4.68		20.11 ± 2.31		25.05 ± 6.10	
Age (years old)			0.166		0.880		0.005
60–69	354 (72.54)	10.88 ± 4.76		20.12 ± 2.31		25.56 ± 5.93	
≥70	134 (27.46)	10.28 ± 4.44		20.07 ± 2.28		23.68 ± 6.35	
Gender			0.834		0.284		0.005
Male	145 (29.71)	10.68 ± 4.51		19.95 ± 2.13		23.76 ± 6.53	
Female	343 (70.29)	10.74 ± 4.76		20.17 ± 2.37		25.59 ± 5.83	
Residence			<0.001		0.068		<0.001
Rural	178 (36.48)	8.65 ± 4.49		19.84 ± 2.20		22.97 ± 5.89	
Urban	213 (43.65)	12.63 ± 4.24		20.40 ± 2.44		26.80 ± 5.92	
Suburban	97 (19.88)	10.32 ± 4.23		19.94 ± 2.11		25.01 ± 5.69	
Education			<0.001		0.082		<0.001
Primary school or below	169 (34.63)	8.60 ± 4.53		19.81 ± 2.06		23.65 ± 5.32	
Junior high school	140 (28.69)	11.26 ± 4.53		20.23 ± 2.44		25.30 ± 6.50	
High school/technical school	98 (20.08)	12.32 ± 4.24		20.07 ± 2.32		25.56 ± 7.01	
Associate degree	58 (11.89)	12.00 ± 4.21		20.31 ± 2.16		27.08 ± 4.45	
Bachelor’s degree or above	23 (4.71)	12.91 ± 4.03		21.21 ± 3.07		26.47 ± 6.51	
Type of employment			<0.001		0.005		<0.001
Long-term stable job (permanent employment)	34 (6.97)	11.88 ± 3.85		20.11 ± 2.53		24.52 ± 5.82	
Temporary job	51 (10.45)	10.37 ± 4.89		19.66 ± 2.38		24.90 ± 6.01	
Freelance	39 (7.99)	12.71 ± 5.01		19.74 ± 2.24		26.23 ± 6.35	
Unemployed	113 (23.16)	7.94 ± 3.63		19.75 ± 2.14		23.46 ± 5.41	
Retired	197 (40.37)	12.06 ± 4.37		20.61 ± 2.29		26.28 ± 6.24	
Other	54 (11.07)	9.79 ± 4.99		19.68 ± 2.25		23.46 ± 6.10	
Household income per capita (CNY)			<0.001		0.018		0.003
<2,000	124 (25.41)	9.63 ± 4.90		19.82 ± 2.17		23.70 ± 6.44	
2,000–4,999	220 (45.08)	10.59 ± 4.69		19.95 ± 2.23		24.85 ± 6.24	
5,000–9,999	126 (25.82)	12.02 ± 4.12		20.59 ± 2.49		26.57 ± 5.25	
≥10,000	18 (3.69)	10.66 ± 4.85		20.66 ± 2.27		25.88 ± 5.42	
Duration of rheumatoid arthritis (Years)			0.008		0.001		0.055
<1	49 (10.04)	8.55 ± 4.37		19.73 ± 1.96		23.46 ± 6.56	
1–3	95 (19.47)	10.68 ± 4.62		19.76 ± 2.20		23.66 ± 7.03	
3–5	100 (20.49)	11.34 ± 4.77		19.84 ± 2.43		24.96 ± 6.39	
5–10	111 (22.75)	11.26 ± 4.33		19.93 ± 2.32		25.78 ± 5.40	
>10	133 (27.25)	10.63 ± 4.86		20.84 ± 2.25		26.07 ± 5.26	
Family member with rheumatoid arthritis			0.007		0.023		0.065
Yes	80 (16.39)	9.46 ± 4.70		19.56 ± 2.20		23.73 ± 6.56	
No	408 (83.61)	10.97 ± 4.64		20.21 ± 2.31		25.30 ± 5.98	
Stage of rheumatoid arthritis			<0.001		0.160		<0.001
Early stage	72 (14.75)	9.23 ± 4.40		19.88 ± 1.85		23.36 ± 6.20	
Active stage	63 (12.91)	10.74 ± 4.64		19.61 ± 2.30		24.11 ± 6.95	
Remission stage	218 (44.67)	11.99 ± 4.54		20.28 ± 2.51		26.63 ± 5.51	
Unsure	135 (27.66)	9.459 ± 4.53		20.17 ± 2.14		23.82 ± 5.98	
Type of medical insurance			<0.001		<0.001		<0.001
Provincial insurance	39 (7.99)	13.41 ± 3.96		21.12 ± 2.24		28.35 ± 5.02	
Municipal insurance	195 (39.96)	12.17 ± 4.51		20.58 ± 2.52		25.85 ± 6.32	
New rural cooperative medical scheme	238 (48.77)	9.12 ± 4.24		19.63 ± 1.98		23.69 ± 5.78	
Other	16 (3.28)	10.31 ± 6.09		18.93 ± 2.20		27.31 ± 5.26	

### Distribution of responses to KAP

The distribution of knowledge responses showed that the three questions with the highest proportion of “Do not understand” responses were: “Common self-assessment tools for RA patients include the Visual Analogue Scale, Disease Activity Score-28, Health Assessment Questionnaire, and Patient Health Questionnaire-9” (K9) with 50.82%, “At the time of your initial diagnosis of RA, your level of understanding of the disease and treatment options” (K5) with 43.85%, and “Children of RA patients may also develop the condition” (K3) with 36.07% (Table 2).

**Table 2:** Distribution of knowledge dimension responses.

Items, *N* (%)	Understand	Partially understand	Do not understand
1. The main symptoms of rheumatoid arthritis include joint swelling and pain, which can lead to joint damage and deformity.	221 (45.29)	231 (47.34)	36 (7.38)
2. Rheumatoid arthritis may be associated with cardiovascular, pulmonary, and skin diseases.	135 (27.66)	256 (52.46)	97 (19.88)
3. Children of rheumatoid arthritis patients may also develop the condition.	95 (19.47)	217 (44.47)	176 (36.07)
4. The treatment goals and evaluation methods for rheumatoid arthritis should be jointly decided by the doctor and the patient.	174 (35.66)	247 (50.61)	67 (13.73)
5. At the time of your initial diagnosis of rheumatoid arthritis, your level of understanding of the disease and treatment options was:	81 (16.6)	193 (39.55)	214 (43.85)
6. Medication treatment for rheumatoid arthritis may have side effects, such as gastrointestinal discomfort and liver function damage.	179 (36.68)	263 (53.89)	46 (9.43)
7. Self-management for rheumatoid arthritis includes exercise, self-assessment, and dietary management.	144 (29.51)	302 (61.89)	42 (8.61)
8. Rheumatoid arthritis patients should engage in light to moderate exercise, such as walking and swimming.	158 (32.38)	268 (54.92)	62 (12.7)
9. Common self-assessment tools for rheumatoid arthritis patients include the Visual Analogue Scale, Disease Activity Score-28, Health Assessment Questionnaire, and Patient Health Questionnaire-9.	52 (10.66)	188 (38.52)	248 (50.82)
10. Rheumatoid arthritis patients should quit smoking and limit alcohol consumption, avoiding high-sugar, trans-fat-containing, and fried foods.	157 (32.17)	276 (56.56)	55 (11.27)

Responses to the attitude dimension showed that 41.6% strongly agreed and 52.05% agreed that RA had significantly negatively impacted their life (A1). In contrast, 11.89% strongly agreed and 29.51% agreed that treatment goals for RA should be set by the doctor alone, and the patient does not need to be involved (A5). Meanwhile, 37.3% disagreed, and 10.66% strongly disagreed that RA can be completely cured (A3) (Table 3).

**Table 3:** Distribution of attitude dimension responses.

Items, *N* (%)	Strongly agree	Agree	Neutral	Disagree	Strongly disagree
1. I believe rheumatoid arthritis has significantly negatively impacted my life.	203 (41.6)	254 (52.05)	13 (2.66)	15 (3.07)	3 (0.61)
2. I believe it is important to comprehensively understand rheumatoid arthritis for its treatment and prognosis.	159 (32.58)	286 (58.61)	33 (6.76)	7 (1.43)	3 (0.61)
3. I believe rheumatoid arthritis can be completely cured.	44 (9.02)	190 (38.93)	20 (4.1)	182 (37.3)	52 (10.66)
4. I am satisfied with the doctor’s decision-making in the diagnosis and management of rheumatoid arthritis.	126 (25.82)	309 (63.32)	29 (5.94)	20 (4.1)	4 (0.82)
5. I believe the treatment goals for rheumatoid arthritis should be set by the doctor alone, and the patient does not need to be involved.	58 (11.89)	144 (29.51)	52 (10.66)	186 (38.11)	48 (9.84)
6. I am confident in managing rheumatoid arthritis on my own.	104 (21.31)	334 (68.44)	25 (5.12)	23 (4.71)	2 (0.41)

Responses to the practice dimension showed that 32.79% occasionally and 19.88% never participated in courses on managing RA (P6). Additionally, 23.36% occasionally and 3.48% never engaged in light-to-moderate exercise, such as walking and swimming (P4), and 21.11% occasionally and 2.25% never quit smoking, limited alcohol, and avoided high-sugar, trans-fat-containing, and fried foods (P5) (Table 4).

**Table 4:** Distribution of practice dimension responses.

Items, *N* (%)	Always	Often	Sometimes	Occasionally	Never
1. I follow the doctor’s advice for treating rheumatoid arthritis.	160 (32.79)	213 (43.65)	45 (9.22)	66 (13.52)	4 (0.82)
2. I pay attention to side effects during the treatment process.	138 (28.28)	188 (38.52)	67 (13.73)	85 (17.42)	10 (2.05)
3. If symptoms worsen, I seek medical attention promptly.	153 (31.35)	190 (38.93)	66 (13.52)	74 (15.16)	5 (1.02)
4. I engage in light to moderate exercises, such as walking and swimming.	95 (19.47)	176 (36.07)	86 (17.62)	114 (23.36)	17 (3.48)
5. I quit smoking, limit alcohol, and avoid high-sugar, trans-fat-containing, and fried foods.	94 (19.26)	184 (37.7)	96 (19.67)	103 (21.11)	11 (2.25)
6. I participate in courses on managing rheumatoid arthritis.	45 (9.22)	108 (22.13)	78 (15.98)	160 (32.79)	97 (19.88)
7. I attend regular follow-up visits and consultations.	174 (35.66)	186 (38.11)	43 (8.81)	73 (14.96)	12 (2.46)

### Correlations between KAP

Correlation analysis revealed positive correlations between knowledge scores and attitude scores (*r* = 0.1293, *p* = 0.0042), as well as between knowledge scores and practice scores (*r* = 0.5037, *p* < 0.001). Additionally, attitude scores were positively correlated with practice scores (*r* = 0.2066, *p* < 0.001) (Supplementary Table S2).

### Measurement model assessment

**Figure 1:**
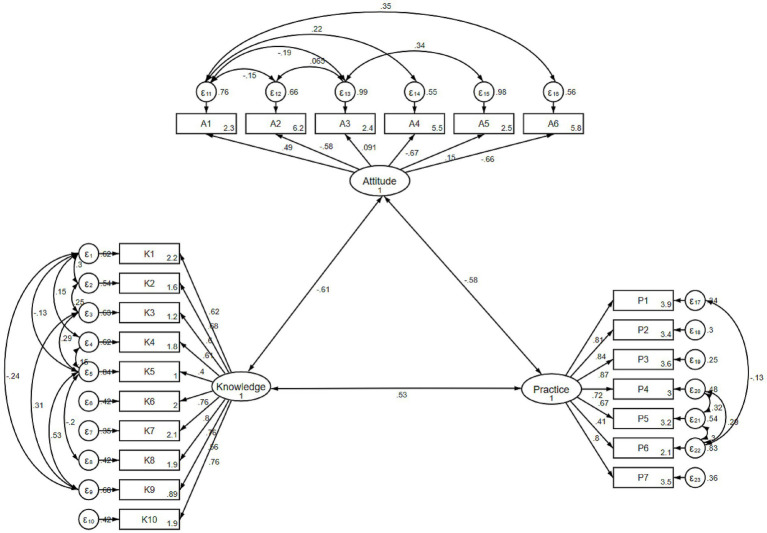
Confirmatory factor analysis of the KAP measurement model.

The KAP questionnaire demonstrated good internal consistency, with a Cronbach’s *α* coefficient of 0.812. The reliability and validity of the measurement model were further assessed using CR, AVE, and the Fornell–Larcker criterion. The Knowledge and Practice constructs demonstrated acceptable internal consistency and convergent validity (CR > 0.80), whereas the Attitude construct showed relatively low reliability and convergent validity (CR = 0.46; AVE = 0.14). Detailed reliability and convergent validity indices are presented in (Supplementary Table S3). Discriminant validity was examined by comparing the square root of AVE for each construct with the inter-construct correlation matrix. The Knowledge and Practice constructs met the Fornell–Larcker criterion, while the Attitude construct showed limited discriminant validity, consistent with its relatively low convergent validity (Supplementary Table S4). CFA was conducted to examine the factor structure of the KAP measurement model (Figure 1). The overall CFA model demonstrated acceptable fit indices (RMSEA = 0.080, SRMR = 0.081, TLI = 0.860, CFI = 0.886), supporting the general adequacy of the three-factor measurement framework (Table 5).

**Table 5:** Model fit indices for the confirmatory factor analysis.

Indicators	Reference	Results
RMSEA	<0.08 Good	0.080
SRMR	<0.08 Good	0.081
TLI	>0.8 Acceptable	0.860
CFI	>0.8 Acceptable	0.886

### SEM analysis

**Figure 2:**
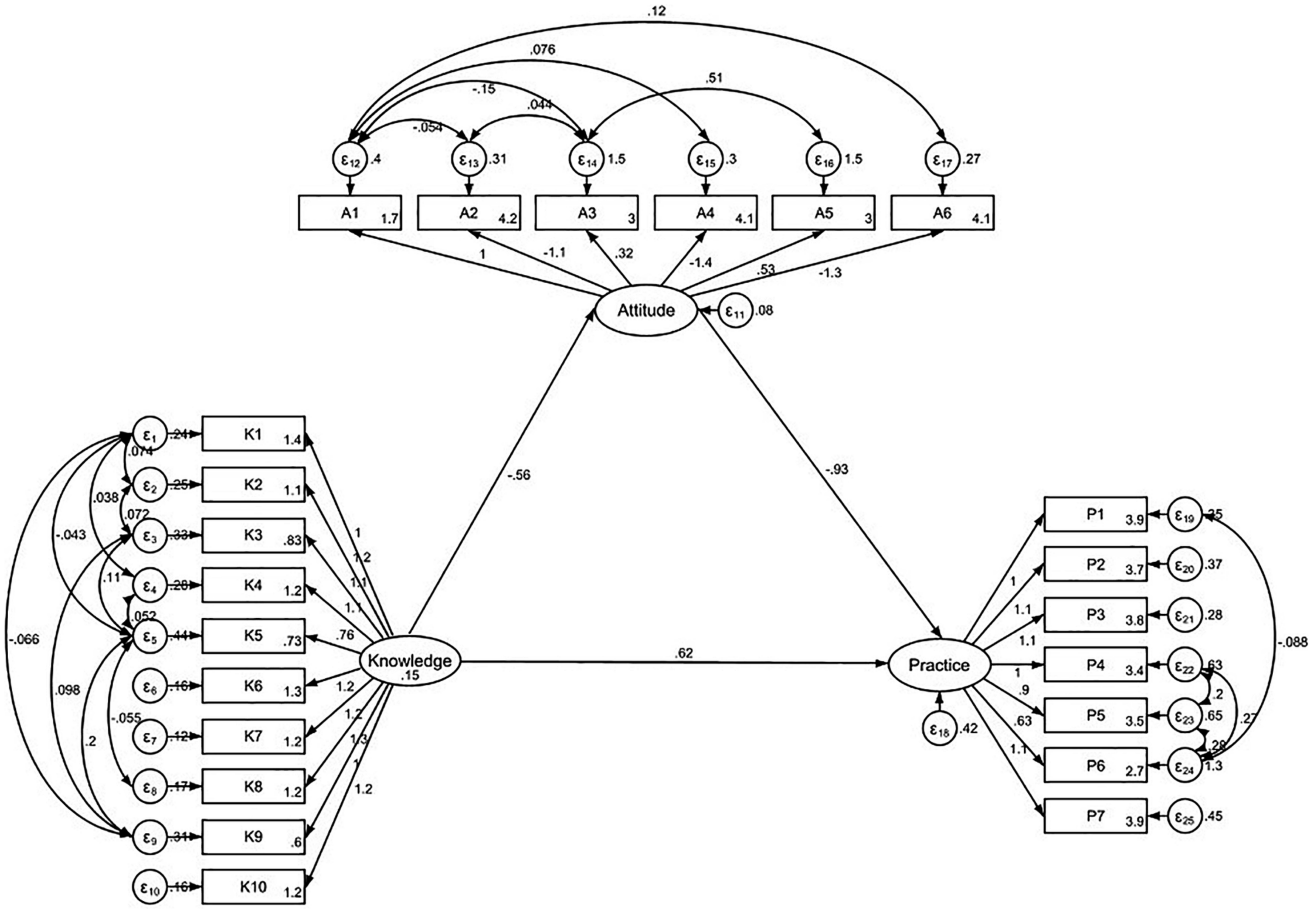
SEM analysis.

The fit of the SEM model yielded acceptable fit indices (RMSEA value: 0.080, SRMR value: 0.081, TLI value: 0.860, and CFI value: 0.886) (Supplementary Table S5). The results showed that knowledge was significantly associated with attitude (*β* = −0.56, *p* < 0.001) and practice (*β* = 0.61, *p* < 0.001). Attitude also directly affected practice (*β* = −0.93, *p* < 0.001). Additionally, knowledge indirectly affected practice through attitude (*β* = 0.52, *p* < 0.001) (Table 6 and Figure 2).

**Table 6:** Direct and indirect effects among latent constructs in the SEM.

Model paths	Total effects		Direct effect		Indirect effect	
	*β* (95% CI)	*P*	*β* (95% CI)	*P*	*β* (95% CI)	*P*
A ← K	−0.56 (−0.73,-0.39)	<0.001	−0.56 (−0.73,-0.39)	<0.001		
P ← A	−0.93 (−1.36,-0.49)	<0.001	−0.93 (−1.36,-0.49)	<0.001		
P ← K	1.14 (0.90,1.38)	<0.001	0.61 (0.34,0.89)	<0.001	0.52 (0.31,0.73)	<0.001

K, Knowledge; A, Attitude; P, Practice. All variables represent latent constructs measured by questionnaire items.

## Discussion

In this study, older adults patients with RA showed generally insufficient knowledge and predominantly negative attitudes, while many still reported relatively proactive disease management behaviors. SEM analysis showed that among older adults RA patients, knowledge significantly impacted both attitudes and practices, with attitudes mediating the relationship between knowledge and practice behaviors. These findings highlight the critical need for comprehensive educational interventions specifically designed for older adults RA patients to improve their knowledge and attitude, which could further enhance disease management outcomes.

Responses to the knowledge dimension revealed substantial gaps among older adults RA patients, particularly in understanding self-assessment methods and disease monitoring. Studies focusing on RA patients have shown that limited disease-specific knowledge directly impacts treatment adherence and self-management capabilities (24, 25). For instance, a hospital-based cross-sectional survey conducted at JiuJiang No. 1 People’s Hospital in eastern China in 2024, involving 504 RA patients, reported that the mean KAP scores for dietary management were 10.13 ± 3.58 (range 0–22), 31.38 ± 2.38, and 4.46 ± 2.30 (range 0–12), respectively, with significant positive correlations between knowledge and practice (*r* = 0.294, *p* < 0.001) and between attitude and practice (*r* = 0.178, *p* < 0.001) (26). Previous research on older adults RA patients has similarly identified significant knowledge gaps regarding disease mechanisms and treatment options. For example, one cross-sectional study conducted in South Korea among 312 older adults RA patients in outpatient settings found that less than 45% could correctly identify disease progression indicators, while a study in northern China involving 276 community-dwelling older adults patients reported insufficient understanding of early treatment goals and medication side effects (27, 28). The attitude dimension showed predominantly negative perceptions among older adults RA patients, with many expressing skepticism about their ability to manage the disease effectively. This aligns with findings from studies specifically examining older adults RA populations, where negative attitudes toward disease management were commonly associated with poor treatment adherence (29, 30). Similar patterns of demographic influence on medication adherence were also reported in a recent study of older adults rheumatic patients in Turkey (31). In terms of practice, our study found that while older adults RA patients generally adhered to medication regimens, they showed less consistent engagement in crucial self-management activities such as exercise and dietary modifications. Similar patterns have been observed in other studies of older adults RA populations. The lower participation in rehabilitation programs and self-management courses among the study participants reflects barriers commonly faced by older adults RA patients (32, 33).

Our comprehensive analysis of demographic characteristics revealed complex patterns in how various factors influenced KAP among older adults RA patients. Geographic and economic factors significantly shaped disease management practices, with rural residents and those with lower household incomes showing less engagement in recommended self-management behaviors. These findings align with previous observations that access to specialized care and rehabilitation services often varies by location (34). Community-based interventions have shown promise in addressing these disparities among older adults RA patients (35, 36). A clear educational gradient was evident, where higher educational attainment correlated with better knowledge scores, likely reflecting broader health literacy patterns and improved access to health information. Urban residents consistently demonstrated better knowledge compared to rural residents, indicating persistent disparities in healthcare resources and educational opportunities. Employment status further emerged as a critical factor, with retired participants and those in stable employment showing better knowledge and practice scores compared to unemployed individuals, highlighting the role of socioeconomic stability in managing chronic conditions. Insurance type also played a significant role across all KAP dimensions, as participants with provincial insurance exhibited better outcomes than those relying on rural cooperative medical schemes. In China, provincial (or urban employee) insurance schemes, such as the Urban Employee Basic Medical Insurance (UEBMI), typically provide broader coverage, higher reimbursement rates, and better access to specialist services compared to the New Rural Cooperative Medical Scheme (NRCMS), which primarily serves rural residents and often offers more limited benefits. These disparities in healthcare access and financial protection may contribute to differences in knowledge acquisition, healthcare-seeking behavior, and overall disease management among older adults RA patients (37). Studies have shown that while the NRCMS improves basic service utilization, it maintains lower reimbursement rates and leads to substantial out-of-pocket expenditures for rural older adults beneficiaries (38). Moreover, comparative evaluations of UEBMI, URBMI, and NRCMS found that UEBMI ensures the highest level of equity in health-related quality of life and healthcare utilization, whereas NRCMS displays greater pro-rich inequity in access and outcomes (39).

Our SEM results provided valuable insights into the complex relationships among knowledge, attitude, and practice. It is important to note that the positive correlations observed among knowledge, attitude, and practice represent simple, unadjusted bivariate associations. In contrast, the structural equation model estimates conditional effects while simultaneously accounting for multiple relationships and shared variance among latent constructs. After controlling for overlapping variance and indirect pathways in the model, the unique structural path coefficients may differ in direction from bivariate correlations (40, 41). Therefore, the negative paths identified in the SEM do not contradict the correlation analysis, but rather reflect conditional relationships within the multivariable model. Notably, the negative paths observed between knowledge and attitude, and between attitude and practice, differ from the traditional assumptions of the KAP framework (42, 43). In older adults patients with RA, greater disease-related knowledge may heighten awareness of disease chronicity, complications, and long-term treatment burden, which could lead to more cautious, worried, or pessimistic attitudes rather than uniformly positive perceptions (43, 44). Such attitudes may be reflected as lower attitude scores, despite coexisting with more appropriate self-management behaviors. Similarly, more negative emotional attitudes may coexist with stronger practice behaviors driven by symptom burden, long-term treatment routines, and close medical supervision (45, 46). These findings suggest that, in older adults RA populations, attitudes may reflect psychological responses to illness severity rather than motivation for health behaviors, highlighting the need for educational interventions that also address emotional and cognitive coping. Among older adults RA patients, the strong direct effect of knowledge on practice underscores the critical role of disease-specific knowledge as a fundamental driver of self-management behaviors. Overall, these findings challenge simplistic assumptions about the influence of knowledge on disease management and highlight the need for more nuanced educational approaches (47).

To improve outcomes for older adults RA patients, our findings suggest several targeted interventions. First, healthcare providers should implement strategies to enhance medication adherence through simplified regimens and clear communication about treatment benefits. Second, support groups specifically designed for older adults RA patients have shown effectiveness in improving disease management attitudes and practices26. Finally, healthcare systems should consider implementing telemedicine options to improve access to rheumatology care for older adults patients (48, 49).

An important methodological consideration of this study relates to the psychometric properties of the KAP measurement model. Although the Knowledge and Practice constructs demonstrated acceptable reliability and convergent validity, the Attitude construct showed relatively weak psychometric performance, with low composite reliability and average variance extracted. This suggests that the attitude items may capture heterogeneous aspects of patients’ perceptions rather than a single cohesive latent construct. It is important to emphasize that the present questionnaire represents an initial exploratory attempt to assess KAP specifically among older adults patients with rheumatoid arthritis in our regional context. As a preliminary instrument tailored to this population, it provides useful insights into disease management behaviors but requires further refinement. Future large-scale studies should focus on revising and optimizing attitude items, conducting more comprehensive psychometric validation, and confirming the measurement structure in independent samples to enhance the robustness and generalizability of the scale.

This study has several limitations that should be considered when interpreting the findings. First, the cross-sectional design precludes the establishment of causal relationships between KAP, limiting the ability to infer temporal or directional effects. Second, the use of self-administered questionnaires may introduce response bias, as participants might overestimate their knowledge or practice or provide socially desirable answers. Third, although this was a multicenter study, participants were recruited using convenience sampling from hospital outpatient settings, which may bias the sample toward patients already engaged in medical care and limit the representativeness of the findings. Additionally, while the measurement model demonstrated acceptable fit indices and internal consistency, the study did not evaluate comprehensive construct validity metrics such as composite reliability or discriminant validity, as the primary focus was on examining structural relationships rather than scale development. Finally, the study was conducted within a relatively short timeframe and included only older adults patients, which may limit the generalizability of the results to broader populations or individuals with different demographic or clinical characteristics.

## Conclusion

In conclusion, our findings indicate that older adults patients with RA face substantial challenges in disease management, particularly with respect to knowledge acquisition and attitudes toward their condition. Future interventions should focus on providing age-appropriate education programs, enhancing access to specialized care, and addressing the unique barriers faced by older adults RA patients. Implementation of these targeted strategies could significantly improve disease management outcomes in this vulnerable population.

## Data Availability

The original contributions presented in the study are included in the article/Supplementary material, further inquiries can be directed to the corresponding author.
