# Supplementary material for: Knowledge attitude and practice of older adults rheumatoid arthritis patients regarding disease management in a cross-sectional study
Source: Front Public Health. 2026 Mar 9;14:1763566. doi: 10.3389/fpubh.2026.1763566 (PMC13006582; doi:10.3389/fpubh.2026.1763566)
Supplement: Supplementary file 1 [file Table_1.docx]

**Supplementary table 1. Study centers**

| **Study centers** |
| --- |
| West China Hospital of Sichuan University |
| Chengdu Fifth People's Hospital |
| Panzhihua Central Hospital |
| The People's Hospital of Leshan |
| Guang'an People's Hospital |
| Nanchong Central Hospital |
| Mianyang Central Hospital |
| The Third Hospital of Mianyang |
| Deyang People's Hospita |
| The Second People's Hospital of Yibin |

**Supplementary table 2. Correlation analysis**

|  | **Knowledge** | **Attitude** | **Practice** |
| --- | --- | --- | --- |
| **Knowledge** | 1 |  |  |
| **Attitude** | 0.1293 (P=0.0042) | 1 |  |
| **Practice** | 0.5037 (P<0.001) | 0.2066 (P<0.001) | 1 |

**Supplementary table 3. Reliability and convergent validity of the KAP measurement model**

| **Construct** | **Cronbach’s α** | **CR** | **AVE** |
| --- | --- | --- | --- |
| **Knowledge** | 0.891 | 0.890 | **0.450** |
| **Attitude** | **0.513** | **0.460** | **0.143** |
| **Practice** | 0.891 | 0.894 | **0.550** |

**CR**: composite reliability

AVE: average variance extracted

**Supplementary table 4. Discriminant validity using the Fornell–Larcker criterion**

|  | **Knowledge** | **Attitude** | **Practice** |
| --- | --- | --- | --- |
| **Knowledge** | 1.00 | 0.622 | 0.528 |
| **Attitude** | 0.622 | 1.00 | 0.597 |
| **Practice** | 0.528 | 0.597 | 1.00 |

**Supplementary table 5. Goodness-of-Fit Indicators for SEM**

| Indicators | Reference | Results |
| --- | --- | --- |
| RMSEA | <0.08 Good | 0.080 |
| SRMR | <0.08 Good | 0.081 |
| TLI | >0.8 Good | 0.860 |
| CFI | >0.8 Good | 0.886 |

RMSEA: Root Mean Square Error of Approximation

SEM: Structural Equation Modeling

SRMR: Standardized Root Mean Square Residual

TLI: Tucker-Lewis Index
