# Supplementary material for: Knowledge attitude and practice of older adults rheumatoid arthritis patients regarding disease management in a cross-sectional study
Source: Front Public Health. 2026 Mar 9;14:1763566. doi: 10.3389/fpubh.2026.1763566 (PMC13006582; doi:10.3389/fpubh.2026.1763566)
Supplement: Supplementary file 2 [file Table_2.docx]

| Dear Friend:  We are researchers from West China Hospital, Sichuan University, and we sincerely invite you to participate in our study. This research aims to understand rheumatoid arthritis patients' knowledge, attitudes, and practices regarding disease management, providing a basis for formulating scientific early intervention strategies. These strategies may help more people in the future and improve patients' health conditions. Participation in this study is voluntary. If you agree to participate, please review the following instructions:  1. Please complete the questionnaire. There are no right or wrong answers; simply answer truthfully. If you encounter any issues during the process, feel free to contact us. Once completed, kindly submit the questionnaire promptly.  2. This study involves a simple questionnaire survey that will not harm your physical or mental well-being. However, it includes some private information such as your gender and age. We will strictly maintain the confidentiality of your information, so you can fill it out with confidence.  3. As a participant, you have the right to access information related to this study and its progress. If you decide to withdraw from the study, please inform us, and your data will not be included in the research results.  Finally, we sincerely thank you for taking the time to support our scientific research!  □I have been informed and agree to use the collected data for scientific research.  Signature of Consent：  Date of Participation：      Year     Month      Day | | | | | | | | | | | |  |
| --- | --- | --- | --- | --- | --- | --- | --- | --- | --- | --- | --- | --- |
| **Part 1 Basic Information** | | | | | | | | | | | |  |
| 1. **Your age：** years old | | | | | | | | | | | |  |
| 1. **Gender：** | | a. Male  b. Female | | | | | | | | | |  |
| 1. **Place of residence：** | | a. Rural  b. Urban  c. Suburban | | | | | | | | | |  |
| 1. **Education level：** | | a. Primary school or below b. Junior high school c. High school/technical school d. Associate degree e. Bachelor's degree or above | | | | | | | | | |  |
| 1. **Type of employment：** | | a. Long-term stable job (permanent employment) b. Temporary job c. Freelancer d. Unemployed e. Retired f. Other | | | | | | | | | |  |
| 1. **Your household per capita monthly income (in RMB):** | | a.<2000  b.2000-4999  c.5000-9999  d.≥10000 | | | | | | | | | |  |
| 1. **Do you have medical insurance:** | | a. Yes  b. No | | | | | | | | | |  |
| 1. **Duration of rheumatoid arthritis (from initial onset to present):** | | a. <1 year b. 1–3 years c. 3–5 years d. 5–10 years e. >10 years | | | | | | | | | |  |
| 1. **Do your family members have rheumatoid arthritis:** | | a. Yes  b. No | | | | | | | | | |  |
| 1. **What treatments have you undergone after being diagnosed with rheumatoid arthritis? (Multiple choices allowed)** | | a. No treatment b. Medication therapy c. Surgery d. Rehabilitation and physiotherapy e. Other | | | | | | | | | |  |
| 1. **Stage of your rheumatoid arthritis:** | | a. Early stage b. Active stage c. Remission stage d. Unsure | | | | | | | | | |  |
| 1. **Your type of medical insurance:** | | a. Provincial insurance b. Municipal insurance c. New Rural Cooperative Medical Scheme d. Other (please specify): _______ | | | | | | | | | |  |
| **Part 2 Knowledge of Rheumatoid Arthritis and Disease Management** | | | | | | | | | |  |  |  |
| 1. **The main symptoms of rheumatoid arthritis include joint swelling and pain, which can lead to joint damage and deformity.** | | | | | a. understand | | b. partially understand | | c. do not understand | | |  |
| 1. **Rheumatoid arthritis may be associated with cardiovascular, pulmonary, and skin diseases.** | | | | | a. understand | | b. partially understand | | c. do not understand | | |  |
| 1. **Children of rheumatoid arthritis patients may also develop the condition.** | | | | | a. understand | | b. partially understand | | c. do not understand | | |  |
| 1. **The treatment goals and evaluation methods for rheumatoid arthritis should be jointly decided by the doctor and the patient.** | | | | | a. understand | | b. partially understand | | c. do not understand | | |  |
| 1. **At the time of your initial diagnosis of rheumatoid arthritis, your level of understanding of the disease and treatment options was:** | | | | | a. understand | | b. partially understand | | c. do not understand | | |  |
| 1. **Medication treatment for rheumatoid arthritis may have side effects, such as gastrointestinal discomfort and liver function damage.** | | | | | a. understand | | b. partially understand | | c. do not understand | | |  |
| 1. **Self-management for rheumatoid arthritis includes exercise, self-assessment, and dietary management.** | | | | | a. understand | | b. partially understand | | c. do not understand | | |  |
| 1. **Rheumatoid arthritis patients should engage in light to moderate exercise, such as walking and swimming.** | | | | | a. understand | | b. partially understand | | c. do not understand | | |  |
| 1. **Common self-assessment tools for rheumatoid arthritis patients include the Visual Analogue Scale, Disease Activity Score-28, Health Assessment Questionnaire, and Patient Health Questionnaire-9.** | | | | | a. understand | | b. partially understand | | c. do not understand | | |  |
| 1. **Rheumatoid arthritis patients should quit smoking and limit alcohol consumption, avoiding high-sugar, trans-fat-containing, and fried foods.** | | | | | a. understand | | b. partially understand | | c. do not understand | | |  |
| **Part 3 Attitudes Towards Rheumatoid Arthritis and Disease Management** | | | | | | | | | | | | |
| 1. **I believe rheumatoid arthritis has significantly negatively impacted my life.** | | a. strongly agree | b. agree | c. neutral | | d. disagree | | e. strongly disagree | | | | |
| 1. **I believe it is important to comprehensively understand rheumatoid arthritis for its treatment and prognosis.** | | a. strongly agree | b. agree | c. neutral | | d. disagree | | e. strongly disagree | | | | |
| 1. **I believe rheumatoid arthritis can be completely cured.** | | a. strongly agree | b. agree | c. neutral | | d. disagree | | e. strongly disagree | | | | |
| 1. **I am satisfied with the doctor’s decision-making in the diagnosis and management of rheumatoid arthritis.** | | a. strongly agree | b. agree | c. neutral | | d. disagree | | e. strongly disagree | | | | |
| 1. **I believe the treatment goals for rheumatoid arthritis should be set by the doctor alone, and the patient does not need to be involved.** | | a. strongly agree | b. agree | c. neutral | | d. disagree | | e. strongly disagree | | | | |
| 1. **I am confident in managing rheumatoid arthritis on my own.** | | a. strongly agree | b. agree | c. neutral | | d. disagree | | e. strongly disagree | | | | |

| **Part 4 Practices Regarding Rheumatoid Arthritis and Disease Management**  8 or more times out of 10 = Always 5–7 times out of 10 = Often 3–4 times out of 10 = Sometimes 1–2 times out of 10 = Occasionally 0 times out of 10 = Never | | | | | |
| --- | --- | --- | --- | --- | --- |
| 1. **I follow the doctor’s advice for treating rheumatoid arthritis.** | a. always | b. often | c. sometimes | d. occasionally | e. never |
| 1. **I pay attention to side effects during the treatment process.** | a. always | b. often | c. sometimes | d. occasionally | e. never |
| 1. **If symptoms worsen, I seek medical attention promptly.** | a. always | b. often | c. sometimes | d. occasionally | e. never |
| 1. **I engage in light to moderate exercises, such as walking and swimming.** | a. always | b. often | c. sometimes | d. occasionally | e. never |
| 1. **I quit smoking, limit alcohol, and avoid high-sugar, trans-fat-containing, and fried foods.** | a. always | b. often | c. sometimes | d. occasionally | e. never |
| 1. **I participate in courses on managing rheumatoid arthritis.** | a. always | b. often | c. sometimes | d. occasionally | e. never |
| 1. **I attend regular follow-up visits and consultations.** | a. always | b. often | c. sometimes | d. occasionally | e. never |
